# Examining the feasibility of a brief parent intervention designed to promote positive food communication with infants

**DOI:** 10.1186/s40814-023-01328-9

**Published:** 2023-06-03

**Authors:** Lyza Norton, Joy Parkinson, Margaret MacGuinness, Neil Harris, Laura Hart

**Affiliations:** 1 Griffith University, 1 Parklands Drive, Southport, QLD 4222 Australia; 2 Griffith University, 170 Kessels Road, Nathan, QLD 4111 Australia; 3 Australian eHealth Research Centre, CSIRO, Level 7, 296 Herston Road, Herston, QLD 4029 Australia; 4 Gold Coast Hospital and Health Service, 1 Hospital Blvd, Southport, QLD 4215 Australia; 5 Centre for Mental Health, Melbourne School of Population and Global Health, University of Melbourne, 207 Bouverie Street, Carlton, VIC 3010 Australia

**Keywords:** Prevention intervention, Feasibility study, Mixed methods research, Child health, Disordered eating

## Abstract

**Background:**

Few prevention interventions exist focusing on supporting parents to use positive food communication at mealtimes, for the prevention of disordered eating. “Mealtime chatter matters (MCM)” is a brief intervention designed for parents of infants. The intervention was designed in collaboration with child health nurses (CHNs) to be embedded into usual care. The overall aim of this study was to test the feasibility of the intervention through examining the acceptability of the MCM content and resources and the potential impact of the intervention on parents.

**Methods:**

This pilot study utilised a mixed methods approach and took place within a regional child health service in Queensland, Australia (October 2021 to June 2022). Participants were parents of infants attending child health education groups and CHNs. The intervention consisted of a brief education session (including accompanying resources), facilitated by a Paediatric Dietitian. The acceptability of MCM content and resources was assessed by both parents and CHNs via self-reported questionnaires and the potential impact on parents assessed via pre-/post-self-reported questionnaires.

**Results:**

Forty-six parents of infants (aged < 8 months) and six CHNs who hosted the intervention and observed the program’s delivery participated in the study. MCM content and resources were highly acceptable to parents and CHNs, as both qualitative and quantitative data concurred. How the program may have potentially impacted parenting practices was unclear from the survey results and further investigation is required to better understand these. Tangible lessons and opportunities to further test this intervention were clear from current results.

**Conclusion:**

Overall, MCM was acceptable to both parents and CHNs, with the content and resources both being highly valued. Parents reported the content to be informative and engaging and CHNs were keen to have such an intervention available in the future. However, further modification and testing is required of MCM. This feasibility study is an essential first step in supporting parents and CHNs to access an evidence-based intervention with the aim of preventing disordered eating.

**Trial registration:**

Griffith University Human Research Ethics Committee (2021/577) and Gold Coast Hospital and Health Service Human Research Ethics Committee (QGC/76618).

**Supplementary Information:**

The online version contains supplementary material available at 10.1186/s40814-023-01328-9.

## Key messages regarding feasibility

- A prevention intervention focusing on parents of infants, with a focus on food communication at mealtimes is a novel intervention warranting an initial pilot study.
- Both parents and child health nurses were highly accepting of the intervention’s content and resources.
- Further research is required to better understand any potential impact the intervention may have on parenting practices.

## Background

There is a lack of disordered eating preventive interventions with a specific focus on the early years [1]. Disordered eating is a broad term that includes a range of behaviors, such as restrictive eating (“dieting”) and binge eating [2]. These behaviors have the potential to evolve into clinical disorders, including binge eating disorder and anorexia nervosa. Eating disorders have devastating effects on young people and their families [3, 4]. Therefore, understanding which modifiable risk factors are likely to heighten or lower the risk of developing disordered eating is an important consideration when developing preventive interventions.

Parents have the primary influence in the development of children’s eating behaviors [5]. There is an opportunity to support parents to create positive mealtime environments at the very beginning, when infants are first learning to eat solid foods. Family mealtimes are a platform for promoting a range of eating skills and an opportunity for parents to role model positive interactions with food. Langdon-Day and Serpell’s [6] systematic literature review identified family mealtimes as involving strong protective and risk factors for eating disorder development. For example, mealtime communication about healthful eating, rather than weight talk, is shown to be protective [7]. On the other hand, in families with high levels of weight talk and teasing, poor family function or low enjoyment, the protective function of family mealtimes is reversed [8]. The crucial factor necessary for family mealtimes to be protective appears to lie in the positivity and supportiveness of the environment [9]. Research supports the notion that “how” a family interacts is an essential ingredient [10]. Therefore, supporting parents to engage in appropriate communication at mealtimes may be a fundamental step in assisting families to create environments where children can thrive.

Food communication is a contemporary and novel field, without a clear definition. However, viewing it in a broad context may be useful in the consideration of how parents communicate about food. We therefore define it as including *any verbal references about monitoring consumption, restriction, encouragement, negotiations, pressure, value judgements about food, enjoyment, refusal or explanations about the nutrition content or biological processes* [11]. Parents may have difficultly knowing “what” to say to their children during mealtimes. A recent study examined both maternal food talk as it related to their young children’s intake. The study found maternal BMI was negatively associated with food talk and overall conversation [12], suggesting mothers were unsure about “how” to talk about food with their child. This is particularly apparent in a culture where people in larger bodies are stigmatized and their confidence in relating to their children about food may be lacking [13]. Irrespective of weight, when parents do communicate, their words can be detrimental; encouragement to diet has been shown to be associated with the development of disordered eating in children [5]. Clearly, providing guidance about positive food communication warrants further examination.

A significant gap exists in preventive interventions which provide clear and practical guidance for parents about *how* to feed their children, in ways that promote healthful eating and reduce disordered eating behaviors [14]. In particular, the amalgamation of family mealtimes with positive food communication represents an exciting opportunity for the field of prevention, as these parenting practices are likely the most potent of all food practices in promoting healthful eating among children. Importantly, they are yet to be the focus of intervention research and have not been the focus of education provided by primary health services.

Therefore, the aim of this study was to examine the feasibility of a brief prevention intervention focused on mealtime communication for parents of infants. Specifically, the primary objective of this study was to establish feasibility through examining: (1) the acceptability of a brief prevention intervention to both parents and CHNs at community health services, (2) the adequacy of the intervention resources for parents, and (3) the potential impact of the intervention on parent knowledge and feeding practices.

## Methods

Our conceptualization of feasibility was informed by the frameworks of the Medical Research Council [15] on design and evaluation of complex health interventions, and Aschbrenner et al. [16] on mixed methods pilot feasibility studies. The feasibility of the developed intervention was conceptualized as consisting of the acceptability and adequacy of the brief intervention.

### Design

As noted above, there is a dearth of universal disordered eating prevention interventions for parents of young children [1]. In evaluating a novel intervention in the under-serviced research area of disordered eating prevention for parents of infants, it is important to first generate feasibility data from end users to understand their needs, before progressing onto larger trials [16, 17]. It is essential to understand “Can the intervention work within a healthcare service?”, as a foundational step. To this end, we conducted an uncontrolled, repeated measures pilot study to examine the feasibility of the intervention for parents and CHNs. Specific objectives were defined as per guidelines developed by Aschbrenner and colleagues [16] and a range of quantitative and qualitative measures were then selected, see Table 1. Parents were asked to complete a self-report questionnaire before and after exposure to the intervention. Child Health Nurses (CHN) completed a single questionnaire after observing the intervention. The mixed method, convergent design was chosen to gather both quantitative and qualitative data simultaneously. Thus, enabling a more nuanced and comprehensive understanding of the feasibility of the prevention intervention. The reporting of this study was guided by the CONSORT 2010 statement: extension to randomised pilot and feasibility trails [18]. Although our study was not randomised, many of the principles were appropriate for comprehensive reporting.

**Table 1:** Description of measures used pre- and post-intervention for parents and Child Health Nurses (CHNs) to measure feasibility domains

Feasibility domain	Parents	CHNs
Acceptability	• Qualitative feedback on MCM via online questionnaires (post-intervention)	• Qualitative and quantitative feedback on MCM via online questionnaires (post-intervention)
Adequacy of intervention resources	• The Perceived Message Cognition Value Scale (PMCV) [19] • Most important MCM message • Qualitative feedback via online questionnaires (post-intervention)	• Qualitative and quantitative feedback on MCM via online questionnaires (post-intervention)
Potential intervention impact on parenting practices	• Feeding Practices and Structure Questionnaire–Milk Feeding (FPSQ-M)-self-report online questionnaire pre-/post-intervention to measure parent feeding practices • Engagement with MCM strategies (self-report online questionnaire pre/post)	N/A

The study received ethical approval through Griffith University Human Research Ethics Committee (2021/577) and Gold Coast Hospital and Health Service Human Research Ethics Committee (QGC/76618). All participants provided informed consent prior to participation.

### Study setting and participants

This study was conducted within four of the 11 Community Child Health Centers, which are part of the Gold Coast Hospital and Health District in Queensland, Australia. All centers provide primary health care and health promotion education to families of children from birth to school age and are government funded. At the time when this research was being conducted, CHNs facilitated a 4-week education sessions groups targeting new parents in four centres, (1–1.5 h each week) covering the following topics: introduction to solids, sleep and settling, play and development and transitioning to parenting. The sessions are designed for parents of infants, (approximately 3- to 6 months old), typically groups are offered continually. However, due to COVID groups had ceased and selected centers with adequate staffing resumed the groups in the second half of 2021. All families who reside within the Gold Coast Health District were eligible to attend these government funded child health services and any parent who attended the group education session titled “introducing solids” was eligible, as the intervention was added to this group session.

The sample in the current study consisted of parents of infants (< 12 months) who were attending the group education sessions at the four Community Child Health Centers who were offering groups between October 2021 and June 2022. Given the aim of the study was to assess feasibility, formal sample size calculations were not conducted. However, a sample size of 40–50 was nominated, based on practical requirements and pilot sample size recommendations from the literature [20]. In keeping with the nominated sample size, Hennink and Kaiser [21] recommend for qualitative data with homogenous groups (e.g., mothers with infants) saturation is achievable with 9 to 17 participants. Therefore, our sample size is appropriate. Only two centers were facilitating groups at the commencement of the data collection period (due to staffing changes resulting from the pandemic) and given the data collection period was scheduled for 6 months, this sample size was deemed achievable. CHNs involved in the study were those who hosted the intervention at their service and were present to observe the program’s delivery.

### Intervention

The intervention, titled “Mealtime Chatter Matters” (MCM), consisted of a 20-min education session facilitated by a Pediatric Dietitian (the researcher LN), as an “add on” to the regular CHN-led education session “introduction to solids”. LN is an experienced Pediatric Dietitian, who has conducted group education session for over 20 years. A facilitation guide was used to ensure integrity of the education session. The intervention involved a facilitated group discussion (encouraging participants to discuss the four key topic areas, listed below) using a PowerPoint presentation, along with resources consisting of a parent handout (laminated, singe A4 page highlighting the key strategies) and an infant feeding bib displaying the MCM logo.

The content for the MCM education session focused on four key topics, which were constructed from a systematic literature review [14] and refined with CHNs, as part of a co-design study [Study under review]:

1. Care: this topic discussed the benefits of creating a joyful mealtime environment. One of the key strategies suggested for parents was to focus on describing the taste or texture of food, rather than commenting on the volume of food being eaten [22].
2. Share: this topic presented the importance of making mealtimes a social time and provided strategies to encourage parents to eat with their children and include them in the preparation of meals [23], such as putting food into the bowl, for infants.
3. Talk: this topic covered the importance of using mealtimes as an opportunity to connect and talk with children. Suggestions were provided for what to talk about and specifically what to avoid. For example, avoiding “diet talk” and labelling of food as “good and bad” [5].
4. Eat: this topic outlined the division of feeding responsibility, with an emphasis on parents deciding “what” is eaten and “when”, while the child decides “how much” to consume [24].

### Procedure

Parents interested in attending CHN-led Parent Groups (four-week program) contacted Community Child Health and registered their interest, providing basic contact and demographic details via administrative staff. Next, they were re-contacted via email once their child was approximately 3 months old, when they were required to confirm or decline their attendance. The week prior to the first group session they were sent an SMS reminder. After conformation of attendance for the Parent Groups, all parents received an additional SMS, arranged by a research team member (MM). This was sent at least one week prior to the session, with information about the current study. The SMS contained a link to the Patient Information and Consent Form (PICF) and baseline questionnaire, which parents could complete prior to attending the education session.

At the beginning of intervention session, the researcher (LN) provided a brief explanation about the study, thanked those who had already completed the online forms, and provided an opportunity for others to complete, using a QR code which linked to the PICF and baseline questionnaire. All parents who were in attendance received the education session and accompanying resources, regardless of whether they consented to be part of the research or not.

Two weeks after attending an intervention session, parents had two opportunities to complete the post-questionnaire. The first was via an SMS containing a link for the post-questionnaire. The second involved the researcher LN briefly attending the beginning of the regular group session to remind participants about the post-questionnaire and provided a QR code for online access.

CHNs who observed the intervention were emailed an electronic link to the questionnaire within one week of the intervention delivery. All questionnaires were administered using the web-based platform LIMEsurvey.

### Measures

To evaluate the feasibility of the MCM intervention, we focused on examining (1) acceptability of the intervention to parents receiving it and CHNs who would embed the intervention as part of usual service delivery; (2) the adequacy of the intervention resources; and (3) preliminary evidence that may indicated whether the intervention had potential to impact on parents’ knowledge and feeding behaviors, with each of these domains using different measures (see Table 1).

#### Parents

To examine participants’ parental feeding practices before and after the intervention, along with the acceptability of the intervention (including the accompanying resources), the authors developed two self-report questionnaires. See Table 1 for an overview. The following measures were included at baseline:

1. Demographic questions (e.g., date of birth, gender, relationship status, number of children and age of child attending the group), were administered at baseline only.
2. The Feeding Practices and Structure Questionnaire–Milk Feeding (FPSQ-M). This is an 18-item validated tool [25] which measures parental feeding practices, specifically as they relate to parental responsiveness and structure around feeding. The questionnaire was developed recently and based on the original Feeding Practices and Structure Questionnaire (FPSQ) [26] that was designed for older children (> 2 years). The FPSQ-M has been shown to have good internal reliability as per Cronbach’s alpha test, ∝  = 0.087 for “feeding on demand”, ∝  = 0.087 for “using food to calm”, ∝  = 0.071 for “persuasive feeding” and ∝  = 0.087 and ∝  = 0.079 for “parent-led feeding” [25].
3. Four items developed by the research team to measure the frequency of parents’ engagement in the MCM-recommended mealtime strategies, see below in Table 1.

The Post-Intervention Questionnaire included:

1. Sects. 2 and 3 from the Pre-Questionnaire, repeated. (The Feeding Practices and Structure Questionnaire–Milk Feeding (FPSQ-M), 18-items and MCM items 1 to 4.)
2. The Perceived Message Cognition Value Scale (PMCV) [19] was used to inform the questions assessing participants value of the messages provided in the MCM handout. Eight items were used to gather feedback on cognitive challenge (∝ = 0.077) and clarity (∝ = 0.082) (e.g., in relation to the MCM handout: not thought provoking to thought provoking), a seven-point scale was used.
3. Four questions were asked in relation to the importance of the four key topics. These questions were developed by the researchers and used a five-point scale (unimportant to very important). The questions were as follows: (1) how important to you was the message of CARE (express joy when eating together), (2) how important to you was the message SHARE (eat together, sit together, tell stories together), (3) how important to you was the message TALK (use mealtimes to talk together and connect) and (4) how important was the message EAT (You provide, they decide). Additionally, participants were provided with the four domains (Care, Share, Talk and Eat) and asked “which of the four messages is the most important to you to use with your child? Only one answer was able to be selected. This was followed by an open-ended question of “Why did you select that message?”
4. Three open ended questions were included to provide qualitative data. The questions were developed to gain an understanding of participants acceptability of the information session and the handout and any additional comments they would like to offer. These three questions were as follows: (1) please tell us your thoughts on the “Mealtime chatter matters” information session? (e.g., what did you like most, what did you like least, what could be improved?), (2) please tell us your thoughts on the “Mealtime chatter matters” handout? (e.g., What did you like most, what did you like least, what could be improved?), and (3) is there anything else you would like to suggest?

#### Child health nurses

Child health nurses who attended the intervention were requested to complete the following self-reported questionnaire seeking feedback on the MCM education session.

1. Demographic questions (e.g., date of birth, years of practice, full-time or part-time employment).
2. MCM feedback: a four-item questionnaire was developed to gain feedback about the MCM content, resources and relevance for ongoing use in the health service. All items used a five-point scale (e.g., completely appropriate to completely inappropriate) and after each scale was the open-ended question of “Why?”. The questions were as follows: (1) how appropriate was the content of MCM? Why? (2) How appropriate was the handout? Why? (3) How useful do you think the education session would be for the ongoing use in the health service? Why? and (4) Do you think any support or training is required to conduct these sessions? If yes, what would be useful? Last, the following question was asked: please provide any other feedback on the education session and/or resources?

### Analyses

Descriptive statistics (frequencies and percentages) were calculated for parent and CHN demographics. To assess the changes before and after the intervention on the FPSQ-M subscale scores and the four MCM items, paired mean differences with 95% confidence intervals were calculated. Parents’ rating of importance of the key topics were extrapolated by comparing the frequency each of the topics was selected as “the most important”.

Data from the PMCV focusing on the handout, was computed to obtain the means and standard deviation for the responses. MCM feedback was provided by the CHNs and the means and standard deviation for the four five-point questions were computed. All quantitative analyses were completed using SPSS (IBM SPSS, V27). To be included in quantitative analyses, parents needed to complete the repeated items, pre- and post-intervention questionnaires.

Qualitative data from the parent post-intervention questionnaires were analyzed using inductive thematic analysis, informed by Braun and Clarke’s method (2006) the data obtained from four open-ended questions. The question asking “Why” they selected the key topic as most important to them and three questions asking about the acceptability of the MCM session and resources were coded in two phases. Firstly, the responses were read and re-read by LN to become immersed in the data and common responses were grouped together, to establish key themes. A second researcher (JP) then reviewed and verified these groupings. Additionally, the CHNs were asked four “Why” questions provided qualitative data which were analyzed as per the method described for parent qualitative data.

## Results

### Participants

#### Parents

Sixteen MCM sessions were presented across four centers, to a total of 93 parents and 6 different CHNs were hosts.

A total of 46 parents (49.5%) completed both the pre and post questionnaires (out of a potential of 93 across 16 groups). Of these, 44 identified as female (95.7%%) and two identifying as males (4.3%). All participants were aged between 25 and 43 years (*n* = 45, mean age 32.69, SD 3.95). (One participant provided her child’s date of birth, as opposed to their own, *N* = 45) Most participants were married, 32/46 (69.60%) or in a domestic partnership, 13/46 (28.30%). Most 40/46 (87.0%) reported having one child and 97.80% had children aged between 2 and 6 and a half months old at the time of the pre-questionnaire. Postcode data revealed all resided in the Gold Coast region of southeastern Queensland.

#### CHNs

A total of six female CHNs completed post questionnaires, with half reporting full-time and the half part-time employment. Years of practice was between 8 and 40 years (mean 19.66 years, SD 11.67) and half the CHNs had attended an earlier focus group (manuscript under peer review).

### Acceptability

1. *Qualitative feedback *via* online questionnaires: parents*

Qualitative feedback on the “Mealtime chatter matters” information session was provided by 35/46 (76%) of parents. The two main themes generated from the data were “informative and relatable content” and the “engaging presentation”. When describing the content “thought provoking” and “the importance of language at mealtimes” were frequently mentioned. The ability for the information to stimulate critical reflection appears to be a consistent theme, as parents reveal they have greater awareness of the effects of their words at mealtimes.

“Thought provoking. New way at looking at mealtimes and the effect of our words” (Parent 45)

Parents frequently expressed their positivity for the clarity of the information provided and the delivery being “engaging”.

“The speaker was very informative and friendly. She shared stories and asked for input. She confirmed my opinions and own research.” (Parent 14).

2. *Qualitative and quantitative feedback: CHNs*

The five-point scale for the four questions all provided the highest level of five (barring one question which received a 4), indicated a very high degree of relevance, usefulness and importance for the MCM content.

### Qualitative

Examining the reasons provided for the very high scores indicates CHNs believe the “importance of the content” and the need for “early intervention” to be strong factors in giving the scores they did. The theme of CHNs valuing the MCM content to assist them to have conversations early with parents was clearly apparent.

“Vital that we start these conversations with parents sooner rather than later to ensure that children are getting a positive start to their food journey and doing this in partnership with their parents.” (CHN 3)

“Early intervention is key in developing healthy meal habits and language.” (CHN 4).

A variety of answers were provided, in relation to the question about the need for support or training requirements to feel confident to conduct the MCM sessions in the future. Some CHNs were interested in further prompts and to initially co-facilitate with a Dietitian. While others were confident to use the materials to assist them in the future.

“No really, just creating awareness and having a “conversation” with parents. I think this topic starts that “conversation”, when we start thinking about introducing food to our baby” (CHN 1)

### Resource adequacy

1. *PCMV handout: parents*

A total of 45/46 parents responded to all items about the cognitive challenge and clarity of the handout, see Table 2. The mean scores for all items were > 6.00, indicating a very high level of acceptability for all 8 items.

**Table 2:** Mean scores for MCM handout acceptability (*N* = 45)

	^a^Item 1	Item 2	Item 3	Item 4	Item 5	Item 6	Item 7	Item 8
N: Valid	45	45	45	45	45	45	45	45
Missing	1	1	1	1	1	1	1	1
Mean	6.84	6.29	6.40	6.84	6.38	6.82	6.62	6.58
SD	0.56	1.56	0.96	0.77	1.28	0.49	0.72	0.87

Items from the MCM Handout acceptability questionnaire with a possible range of 1 to 7

(^a^See Supplementary information 1 for list of items)

### Qualitative

Feedback on the “Mealtime chatter matters” handout was provided by half the parents (23/46, 50.0%). The main themes identified were “easy to understand” and “reinforced messages”. Many parents reported the handout to be “clear and makes sense”, suggesting it was a practical guide, which they found easy to comprehend. Additionally, there were numerous comments about the use of the handout as being useful “to refresh the memory after the session”. The concept of referring to the handout at a later stage was made with comments such as “Loved the handout, it’s on the fridge for all to read” (Parent 17). These comments implying the handout was both valued and useful.

2. *Most important message: parents*

All parents completed the question “Which of the four messages are the most important to you to use with your child?”. The results were: “Talk” (17/46, 37.0%), “Care” (11/46, 23.90), “Eat” (8/46, 17.4%) and “Share” (7/46, 15.2%).

### Qualitative

The open-ended question “Why did you select that message?” provided parents with the opportunity to reflect on the reason for their selection. “Talk”, was the message selected most, at 37.0%. A strong theme of “creating a positive mealtime environment” was generated as parents expressed a desire to create this for their children, through their communication.

“So that my children will choose to talk to me and express his feeling and share stories during mealtimes, making them a positive experience.” (Parent 16)

3. *Qualitative and quantitative feedback: CHNs*

Feedback from the single question: How appropriate was the handout? Why?

Five out of the six CHN provided the highest score of completely appropriate (5), with the other being appropriate (4). The qualitative data reflected these results, with CHNs revealing they valued the handout as a useful reminder for parents.

“Easy for the parents to refer to as a memory aide and encourage them in the right direction with conversation at mealtimes and supporting their children” CHN3

### Potential impact

1. *FPSQ-M: parents*

Mean differences (with 95% CIs) were calculated to compare the pre- and post-data from subscales of the FPSQ-M questionnaire, see Table 3.

2. *MCM items: parents*

**Table 3:** Mean differences (with 95% CIs) of pre-/post-results for the subscales of the FPSQ-M questionnaire

			Pre-/post-differences				
Subscales	Pre Mean	Post Mean	Mean	SD	Std error mean	95% CI of the difference	
						Lower	Upper
Feeding on demand	4.559	3.701	− 0.886	0.823	0.121	− 1.130	− 0.641
Using food to calm	2.591	2.674	0.826	0.496	0.073	− 0.065	0.230
Parent-led feeding	1.841	1.833	− 0.007	0.525	0.077	− 0.163	0.149
Persuasive feeding	1.877	1.841	− 0.036	0.559	0.082	− 0.202	0.130

Subscales of the Feeding Practices and Structure Questionnaire–Milk (FPSQ-M), with a possible range of one to five [25]

Mean differences (with 95% CIs) were calculated for the pre- and post-results from the MCM items, see Table 4.

**Table 4:** Mean differences (with 95% CIs) of pre-/post-results for the mcm items

			Pre-/post-differences				
MCM item number	Pre Mean	Post Mean	Mean	SD	Std error mean	95% CI of the difference	
						Lower	Upper
1	2.761	3.544	-0.783	1.191	0.176	-1.136	-0.429
2	4.239	4.239	0.000	1.155	0.170	-0.343	0.343
3	4.522	4.174	0.348	0.737	0.109	0.129	0.567
4	4.130	4.109	0.022	1.202	0.177	-0.335	0.379

Items:

1. I talk about the taste or texture of the food during mealtimes

2. During mealtimes I use words like “good” and “bad” to describe foods

3. I enjoy eating with my family and see mealtimes as a time to connect and talk

4. I talk about eating less during mealtimes

## Discussion

Our study used a mixed methods approach, to assess the feasibility of a brief universal intervention. The intervention targeted parents of infants and focused on mealtime communication, for the prevention of disordered eating. This approach is a first step in beginning to answer the call to action from numerous experts in the eating disorder field, who advocate for the use of knowledge translation research within healthcare environments [27, 28]. Our results provide important insights into parent and CHN perspectives that can be used to inform future intervention research.

### Acceptability

Three-quarters of the parents provided qualitative data about the MCM information session, reflecting their desire to provide additional comments. The two main themes generated from the data were “informative and relatable content” and the “engaging presentation”. This positive data from parents, may in part be attributed to the use of the Knowledge to Action Framework (KTA) [29]. Using theory to guide prevention intervention and partnering with communities are argued as two ways to improve the effectiveness of interventions [27, 30]. We used the KTA framework to guide the larger knowledge translation project, with this current study representing the feasibility evaluation phase. The framework suggests first using a knowledge creation cycle to develop an evidence base and then adapting this knowledge for use in specific environments. We chose to use a co-design approach working with CHNs for this step, asking what strategies they felt correlated with the evidence-based strategies provided [article currently under peer review]. Thus, ensuring the intervention was tailored and relevant for those that would ultimately use it.

Our study found CHNs highly valued the MCM content and resources, with both the qualitative and quantitative data reflecting this result. Our findings are consistent with previous research that implemented an evidenced-based program providing preventive strategies for child health nurses to use with parents of children 2–6 years old [31]. This study revealed CHNs valued the content and believed early intervention and prevention of eating problems was a core part of their work in a community child health service [31]. Interestingly, one of the recommendations from CHNs in this study was to start having conversations with parents when their children were younger; when they were transitioning to solids was deemed an appropriate time. One potential reason the content of the MCM education session was likely accepted by the CHNs in our study was they perceived it as valuable and worthwhile. In accordance with Rogers’ theory on the diffusion of innovations, this concept is termed “compatibility” and refers to how closely the innovation aligns with user values, past experiences and needs [32]. This is a critical concept when it comes to busy clinicians, working in demanding environments. For a new innovations to be used in practice, its integration into the system for sustainable uptake is essential. Supporting families with nutrition and eating advice is part of the role of CHNs, therefore having access to evidence-based tools is vital. Our findings suggest that the MCM program was highly acceptable to parents and nurses for delivery in the Child Health Service setting.

### Adequacy

The mean scores for the items related to the MCM Handout acceptability were very high, indicating parents found the handout fit for purpose. Interestingly, the qualitative data revealed some reasons as to “why” they valued the MCM handout. The theme of clear and easy to understand information was highlighted by many parents. The handout was deliberately designed to be a single A4 page of information, a decision adopted directly from insights gained through consulting with CHNs in the co-design of MCM education session [article currently under peer review]. During the co-design, CHNs unanimously expressed their view that a take home resource was required to summarise the MCM session and believed it needed to be “hard copy”, rather than digital. This is in keeping with the literature, which reveals parents are overwhelmed by the vast amounts of child feeding education on the internet [33]. Our study complements these findings as the majority of CHNs reported the parent handout being completely appropriate and no mention of digital options was expressed.

When parents were asked which of the four MCM key messages they valued as the most important to use with their child and why, 37% of parents reported “Talk”. Parents reported it encompassed the other messages and conveyed their desire to create mealtime environments that are calm and joyful. It appears from the qualitative data parents are keen to engage and communicate at mealtimes. They reported feeling validated to connect and talk with their infants during mealtimes, rather than “forcing” them to eat more food. The concept of improved self-efficacy also resonated in the responses to “Eat”. The data associated with “Eat” centered on the importance of letting infants decide on how much to eat and many parents highlighted this strategy contrasted with how they were raised. This represents a potentially important shift in breaking the cycle intergenerationally, as direct associations between parents with disordered eating and their use of unhelpful feeding behaviors have been found in the literature [34]. This finding underscores the opportunity to lay a positive foundation for lifelong habits around food and eating in the early years [35]. Our findings suggest that the parents and nurses found the messages and resources associated with the MCM intervention to be adequate and suited to their needs.

### Potential impact

Our findings related to the potential impact of the MCM intervention on parenting practices were much less clear than findings related to acceptability and adequacy. The “Feeding on demand” subscale of the FPSQ-M scores after the intervention were lower than at pre-test, indicating less “Feeding on demand”. We are unsure why this finding was observed, given it was in the opposite direction to that expected, but offer some possible explanations. First, the *FPSQ-M* questionnaire is relatively new and has not been comprehensively validated. It has, for example, not undergone test-retesting or previously been used in an intervention study. It is therefore possible that the decrease in scores we found over time is an effect of the properties of the measure, rather than the impact of the intervention. However, without a control group, we can only speculate about this.

Another explanation for the result may be in the construct of the items. In the mentioned subscale, half of the four items were reverse coded and perhaps the parents may have responded inappropriately to the change in scale structure across items. Further validation studies, especially with mothers of infants in a busy setting—such as our administration was—is important to rule this out and to ensure the quality of responding among participants.

Two further explanations are also plausible. First, it is possible that the measure did not tap into the constructs MCM was designed to change. Our intervention was developed with a focus on mealtime communication, while the *FPSQ-M* responsiveness subscale was designed to measure the “structure” of infant feeding. Thus, perhaps the measure was not an appropriate fit for our intervention. Alternatively, it is possible that MCM had the undesired effect of reducing responsive feeding. We believe this however, is unlikely, given the high acceptability and adequacy of the intervention outlined above, and the focus of the program on positive mealtime communication, which pre-supposes responsive feeding. Only further research, with a control group and additional, validated and relevant measures, will provide more insights.

To overcome the limitations of the FSPQM, four items that directly mapped onto knowledge and behaviours the MCM intervention was trying to change, were also implemented. Of the four items, three showed small changes in mean scores in the expected direction across the two administrations, but the final item showed a difference in the unexpected direction. On the item about enjoyment of meals, parents reported less enjoyment post intervention compared to pre-intervention. Again, it is unclear why this result was found and it appears to contrast with the positive findings in the acceptability and adequacy data. We suggest four possible explanations. First, the four MCM items were constructed with two being reverse-coded; hence, the result may be erroneous. Second, it is possible that the content of the intervention brought parents’ attention to their engagement or satisfaction with mealtimes in a way that was not salient before the intervention. With time to reflect on the nature of mealtimes, perhaps parents provided a more reflective score at post-intervention than they did at baseline. Previous research supports the notion that mealtimes with infants can be stressful for parents [36], and mealtime enjoyment may be affected by a wide variety of environmental factors not measured in this study (e.g., sleep deprivation, infant tiredness/illness, parental stress, child rejection of foods). Third, recent research has shown parents use a range of feeding behaviours, driven by a variety of parent factors, including mood, and these are not static over time [37]. It is possible that as infants age and come closer to weaning age, that mealtimes become more difficult or stressful and thus less enjoyable for parents; and this may have resulted in our recorded change in scores. It is also possible that daily variability in mealtime enjoyment may be very high, and thus ecological momentary assessment with averaging over more administrations would be a more accurate measure of enjoyment. Last, it is possible the intervention functioned to decrease parents’ enjoyment of mealtimes, perhaps through perceived pressure for mealtimes to be satisfying and joyful. Perhaps the MCM program increased parental expectations of mealtimes being positive, when the reality is often infants can be fussy, messy and unpredictable.

Our findings suggest that whether the MCM intervention has potential to impact on parents feeding behaviours remains unclear and further testing of the resource with robust, validated measures, is required.

## Implications for future research

Given the lack of validated tools available to adequately measure food communication between parents and their children, further research is required prior to retesting of MCM with a pilot group. Perhaps extending existing child feeding questionnaires to include specific questions relating to the language parents use to describe foods (e.g., “good” vs “bad”) would be advantageous. Such measures would then enable a more robust measure of food communication between parents and infants. Additionally, ethnography studies may be useful in broadening our understanding about how parents are communicating about food, within a family mealtime context. There is limited research specifically focused on the content of family mealtime conversations. However, a qualitative study of 150 family groups, conducted by Thomas and colleagues [38], highlighted parents believed it was their job to tell children about the dangers of “fatness” and frequently used negatively framed messages and scare tactics during mealtimes. Hence, the importance of continuing to explore and expand this area of research, as many opportunities exist to further develop interventions to support parents with this essential part of daily life.

## Strengths and limitations

The strengths of this study include the triangulation of data through collection of quantitative and qualitative data to assess feasibility of the intervention. Developing interventions with end users and subsequently piloting them in real world conditions is essential for improving effectiveness. The temptation for researchers is to design complex and costly RCT trials only to discover no health service has the capacity or interest in their ongoing delivery. Hence, a strength of our pilot study was the collection of data from parents and CHNs within a health service setting. We acknowledge this is a brief intervention and not aimed at parents with an eating disorder; however, it is a cost-effective and practical way to provide universal prevention messages. A feasibility study by Sadeh-Sharvit and colleagues [39], examined an intervention aimed at mothers with eating disorders and their spouses, targeted behavioral change in feeding practices, in a small sample (*n* = 16). Findings revealed improved feeding practices; however, the investment was very extensive, as the program required attendance at 12 group sessions (90 min each) followed by a further 12 family sessions (1 h in duration). Given the mean age of the children was 19.6 months, perhaps many of the feeding practices were already established. A further strength of our study was the use of the same facilitator for all groups, increasing the fidelity of the content presented. Additionally, the facilitator was an experienced Pediatric Dietitian able to succinctly provide real life case examples, highlighting the importance of preventive interventions. Despite these strengths, there were several limitations which need to be acknowledged. There was a lack of a control group, therefore causality cannot be assumed from the results of the pre-/post-data. However, the qualitative data provided consistent themes relating to specific MCM strategies and parents’ willingness to implement the recommended strategies. An additional limitation was the lack of a set progression criteria determined prior to the study. Such criteria would have been beneficial to include, enabling set boundaries for moving to a larger trial or modifying the intervention or measures. Examples include, mean acceptability rating of greater than 80% for content and resources, from both CHNs and parents and less than 10% missing data in surveys.

Another limitation was the use of the FPSQ-M questionnaire [25]. While a validated instrument, the items were not directly related to our core content of “mealtime communication” which made it more difficult to draw meaningful outcome conclusions. However, the data provided information on parental feeding behaviors (e.g., feeding on demand, using food to calm) not previously examined in an intervention study targeting infants, and this remains a strength of the current study.

## Conclusions

Findings from this study suggest a brief preventive intervention co-designed with CHNs, with a focus on food communication, is acceptable within a community child health setting. Both parents and CHNs highly valued the content and accompanying resources. Our results, however, suggest further refinements and testing are required to understand whether the MCM intervention has the potential to impact on parent knowledge and behaviors. This further development work would be valuable to complete before embarking on larger trials. Despite some shortcomings, our study found that supporting parents to create positive mealtime experiences was a valued endeavor and should be the focus of future research to help prevent disordered eating in childhood.

## Supplementary Information

**Additional file 1: Supplementary information 1. **The perceived message cognition value scale: items used in post-intervention questionnaire.

## Data Availability

The datasets used during the current study are available from the corresponding author on reasonable request.

## References

[1] Le LK, Barendregt JJ, Hay P, Mihalopoulos C (2017). Prevention of eating disorders: a systematic review and meta-analysis. Clin Psychol Rev.

[2] Goldschmidt AB, Wall M, Choo T-HJ, Becker C, Neumark-Sztainer D. Shared risk factors for mood-, eating-, and weight-related health outcomes. Health Psychol. 2016;35(3):245–52. 10.1037/hea0000283 PMC4760867 26690639

[3] Marzilli E, Cerniglia L, Cimino S (2018). A narrative review of binge eating disorder in adolescence: prevalence, impact, and psychological treatment strategies. Adolesc Health Med Ther.

[4] Herpertz-Dahlmann B, Dahmen B. Children in Need-Diagnostics, Epidemiology, Treatment and Outcome of Early Onset Anorexia Nervosa. Nutrients. 2019;11(8):1932. 10.3390/nu11081932 PMC6722835 31426409

[5] Neumark-Sztainer D, Bauer KW, Friend S, Hannan PJ, Story M, Berge JM. Family weight talk and dieting: how much do they matter for body dissatisfaction and disordered eating behaviors in adolescent girls? J Adolesc Health. 2010;47(3):270–6.e. 10.1016/j.jadohealth.2010.02.001 PMC2921129 20708566

[6] Langdon-Daly J, Serpell L (2017). Protective factors against disordered eating in family systems: A systematic review of research. J Eat Disord.

[7] Berge JM, MacLehose R, Loth KA, Eisenberg M, Bucchianeri MM, Neumark-Sztainer D (2013). Parent conversations about healthful eating and weight: associations with adolescent disordered eating behaviors. JAMA Pediatr.

[8] Loth K, Wall M, Choi CW, Bucchianeri M, Quick V, Larson N (2015). Family meals and disordered eating in adolescents: are the benefits the same for everyone?. Int J Eat Disord.

[9] Dallacker M, Hertwig R, Mata J (2019). Quality matters: a meta-analysis on components of healthy family meals. Health Psychol.

[10] Middleton G, Golley R, Patterson K, Le Moal F, Coveney J. What can families gain from the family meal? A mixed-papers systematic review. Appetite. 2020:104725. 10.1016/j.appet.2020.104725 32422173

[11] Roach E, Viechnicki GB, Retzloff LB, Davis-Kean P, Lumeng JC, Miller AL (2017). Family food talk, child eating behavior, and maternal feeding practices. Appetite.

[12] DeJesus JM, Gelman SA, Viechnicki GB, Appugliese DP, Miller AL, Rosenblum KL (2018). An investigation of maternal food intake and maternal food talk as predictors of child food intake. Appetite.

[13] Tomiyama AJ, Carr D, Granberg EM, Major B, Robinson E, Sutin AR (2018). How and why weight stigma drives the obesity 'epidemic' and harms health. BMC Med.

[14] Norton L, Parkinson J, Harris N, Darcy M, Hart L. Parental food communication and child eating behaviours: a systematic literature review. Health Promot J Austr. 2022. 10.1002/hpja.604. 10.1002/hpja.604 35363899

[15] Craig P, Dieppe P, Macintyre S, Michie S, Nazareth I, Petticrew M (2008). Developing and evaluating complex interventions: the new Medical Research Council guidance. BMJ.

[16] Aschbrenner KA, Kruse G, Gallo JJ, Plano Clark VL. Applying mixed methods to pilot feasibility studies to inform intervention trials. Pilot Feasibility Stud. 2022;8(1):217. 10.1186/s40814-022-01178-x PMC9511762 36163045

[17] Campbell NC, Murray E, Darbyshire J, Emery J, Farmer A, Griffiths F (2007). Designing and evaluating complex interventions to improve health care. Br Med J.

[18] Eldridge SM, Chan CL, Campbell MJ, Bond CM, Hopewell S, Thabane L, et al. CONSORT 2010 statement: extension to randomised pilot and feasibility trials. BMJ. 2016:355. 10.1136/bmj.i5239 PMC5076380 27777223

[19] Lane DR, Grant Harrington N, D’onohew L, Zimmerman RS (2006). Dimensions and validation of a perceived message cognition value scale. Commun Res Rep.

[20] Julious SA (2005). Sample size of 12 per group rule of thumb for a pilot study. Pharm Stat.

[21] Hennink M, Kaiser BN (2022). Sample sizes for saturation in qualitative research: a systematic review of empirical tests. Soc Sci Med.

[22] Loth KA (2016). Associations between food restriction and pressure-to-eat parenting practices and dietary intake in children: a selective review of the recent literature. Curr Nutr Rep.

[23] Robson SM, McCullough MB, Rex S, Munafo MR, Taylor G (2020). Family meal frequency, diet, and family functioning: a systematic review with meta-analyses. J Nutr Educ Behav.

[24] Black MM, Aboud FE (2011). Responsive feeding is embedded in a theoretical framework of responsive parenting. J Nutr.

[25] Jansen E, Russell CG, Appleton J, Byrne R, Daniels LA, Fowler C (2021). The Feeding Practices and Structure Questionnaire: development and validation of age appropriate versions for infants and toddlers. Int J Behav Nutr Phys Act.

[26] Jansen E, Mallan KM, Nicholson JM, Daniels LA (2014). The feeding practices and structure questionnaire: construction and initial validation in a sample of Australian first-time mothers and their 2-year olds. Int J Behav Nutr Phys Act.

[27] Austin SB (2016). Accelerating progress in eating disorders prevention: a call for policy translation research and training. Eat Disord.

[28] Neumark-Sztainer D (2016). Eating disorders prevention: looking backward, moving forward; looking inward, moving outward. Eat Disord.

[29] Graham ID, Logan J, Harrison MB, Straus SE, Tetroe J, Caswell W (2006). Lost in knowledge translation: Time for a map?. J Contin Educ Health Prof.

[30] Ciao AC, Loth K, Neumark-Sztainer D (2014). Preventing eating disorder pathology: Common and unique features of successful eating disorders prevention programs. Curr Psychiatry Rep.

[31] Norton LN, Hart LM, Butel FE, Roberts S (2020). Child health nurse perceptions of using confident body, confident child in community health: a qualitative descriptive study. BMC Nurs..

[32] Rogers EM (2002). Diffusion of preventive innovations. Addict Behav.

[33] Holmberg Fagerlund B, Helseth S, Glavin K (2019). Parental experience of counselling about food and feeding practices at the child health centre: a qualitative study. J Clin Nurs.

[34] Norton L, Parkinson J, Harris N, Hart LM. What factors predict the use of coercive food parenting practices among mothers of young children? An examination of food literacy, disordered eating and parent demographics. Int J Environ Res Public Health. 2021;18(19):10538. 10.3390/ijerph181910538 PMC8508140 34639838

[35] Montano Z, Smith JD, Dishion TJ, Shaw DS, Wilson MN (2015). Longitudinal relations between observed parenting behaviors and dietary quality of meals from ages 2 to 5. Appetite.

[36] Matvienko-Sikar K, Kelly C, Sinnott C, McSharry J, Houghton C, Heary C (2018). Parental experiences and perceptions of infant complementary feeding: a qualitative evidence synthesis. Obes Rev.

[37] Loth KA, Ji Z, Wolfson J, Neumark-Sztainer D, Berge JM, Fisher JO (2022). A descriptive assessment of a broad range of food-related parenting practices in a diverse cohort of parents of preschoolers using the novel Real-Time Parent Feeding Practices Survey. Int J Behav Nutr Phys Act.

[38] Thomas SL, Olds T, Pettigrew S, Randle M, Lewis S (2014). "Don't eat that, you'll get fat!" Exploring how parents and children conceptualise and frame messages about the causes and consequences of obesity. Soc Sci Med.

[39] Sadeh-Sharvit S, Zubery E, Mankovski E, Steiner E, Lock JD (2016). Parent-based prevention program for the children of mothers with eating disorders: Feasibility and preliminary outcomes. Eat Disord.

