# Supplementary material for: Examining the feasibility of a brief parent intervention designed to promote positive food communication with infants
Source: Pilot Feasibility Stud. 2023 Jun 3;9:93. doi: 10.1186/s40814-023-01328-9 (PMC10239088; doi:10.1186/s40814-023-01328-9)
Supplement: Supplementary file 1 — Additional file 1: Supplementary information 1. The perceived message cognition value scale: items used in post-intervention questionnaire. [file 40814_2023_1328_MOESM1_ESM.docx]

**Supp 1.**

*The perceived message cognition value scale: items used in post-intervention questionnaire*

| **Item** | **1** | **2** | **3** | **4** | **5** | **6** | **7** |  |
| --- | --- | --- | --- | --- | --- | --- | --- | --- |
| 1.Not understandable at all | ☐ | ☐ | ☐ | ☐ | ☐ | ☐ | ☐ | Very understandable |
| 2.*Would make people think |  |  |  |  |  |  |  | Would not make people think |
| 3. Did not help me know what to do |  |  |  |  |  |  |  | Really helped me to know what to do |
| 4. Does not make sense |  |  |  |  |  |  |  | Makes sense |
| 5. Not at all thought-provoking |  |  |  |  |  |  |  | Thought-provoking |
| 6. Unclear information presented |  |  |  |  |  |  |  | Clear information presented |
| 7. Did not really make me think |  |  |  |  |  |  |  | Really made me think |
| 8. Did not provide a clear call to action |  |  |  |  |  |  |  | Really provides a clear call to action |

*= reverse coded
